# Caffeic Acid Phenethyl Ester Inhibits UV-Induced MMP-1 Expression by Targeting Histone Acetyltransferases in Human Skin

**DOI:** 10.3390/ijms20123055

**Published:** 2019-06-22

**Authors:** Eun Ju Shin, Seongin Jo, Hyo-kyoung Choi, Sungbin Choi, Sanguine Byun, Tae-Gyu Lim

**Affiliations:** 1 Korea Food Research Institute, Wanju-gun, Jeollabuk-do 55365, Korea; Shin.Eun-Ju@kfri.re.kr (E.J.S.); chkyoung@kfri.re.kr (H.-k.C.); 2 Division of Bioengineering, Incheon National University, Incheon 22012, Korea; friend9698@naver.com (S.J.); 201921148@inu.ac.kr (S.C.)

**Keywords:** caffeic acid phenethyl ester, MMP-1, skin aging, histone acetylation, histone acetyltransferase

## Abstract

Caffeic acid phenethyl ester (CAPE), a naturally occurring bioactive compound, displays anti-inflammatory, anti-carcinogenic, and anti-microbial effects. However, the effect of CAPE on skin photoaging is unknown. Herein, we investigated the inhibitory effect of CAPE against ultraviolet (UV) irradiation-mediated matrix metalloproteinase (MMP)-1 expression and its underlying molecular mechanism. CAPE treatment suppressed UV-induced MMP-1 levels in both human dermal fibroblasts (HDF) and human skin tissues. While CAPE did not display any significant effects against the upstream regulatory pathways of MMP-1, CAPE was capable of reversing UV-mediated epigenetic modifications. CAPE suppressed UV-induced acetyl-histone H3 (Lys9) as well as total lysine acetylation in HDF cells. Similarly, CAPE also attenuated UV-induced lysine acetylations in human skin tissues, suggesting that the CAPE-mediated epigenetic alterations can be recapitulated in ex vivo conditions. CAPE was found to attenuate UV-induced histone acetyltransferase (HAT) activity in HDF. Notably, CAPE was able to directly inhibit the activity of several HATs including p300, CREP-binding protein (CBP), and p300/CBP-associated factor (PCAF), further confirming that CAPE can function as an epigenetic modulator. Thus, our study suggests that CAPE maybe a promising agent for the prevention of skin photoaging via targeting HATs.

## 1. Introduction

Photoaging is mainly caused by ultraviolet (UV) exposure, free radicals, and physical stimuli [1]. The UV light reaching our skin is composed of 90–95% UVA (315–400 nm) and 5–10% UVB (280–315 nm). Overexposure to UVA/UVB is known to be associated with skin aging characterized by wrinkle formation, skin pigmentation, and laxity [2,3]. UV irradiation causes the expression of various matrix metalloproteinases (MMPs) in dermal fibroblasts, which contribute to the degradation of extracellular matrixes including collagen, leading to skin wrinkle formation [4]. Among the UV-inducible enzymes, MMP-1 has been known to play a key role in collagen degradation, and thus has been a major interest in skin aging [4,5]. Therefore, MMP-1 inhibitors can be an attractive strategy in preventing skin aging due to UV exposure.

Recent reports have suggested a critical link between UV-mediated epigenetic modifications and skin aging [3]. Histone modification plays a key role in chromatin restructuring and the regulation of gene transcription [6]. Acetylation of histone proteins is reversibly regulated by two major groups of enzymes, the histone acetyltransferases (HATs) and the histone deacetylase (HDACs), which regulate the transfer of acetyl groups to the lysine side chain of histone proteins [7]. UV-mediated induction of MMP-1 is associated with hyperacetylation of histone H3 mediated by HATs including p300 [8,9]. These studies provide the rationale that agents controlling HAT activity might be useful for the transcriptional regulation of MMP-1.

Caffeic acid phenethyl ester (CAPE) is one of the major components of natural products obtained from propolis and other plants such as *Rhodiola sacra* and *Euonymus alatus* [10,11,12]. CAPE is known to have important biological activities such as antioxidant, anti-inflammatory, and anti-cancer properties [13,14]. However, the protective effects of CAPE against UV-induced skin photoaging have not yet been reported. In this study, we investigated the effects of CAPE on UV-induced skin aging in both HDF cells and human skin tissue and examined the underlying molecular mechanism responsible.

## 2. Results

### 2.1. CAPE Reduces UV-Induced MMP-1 Expression in Human Dermal Fibroblasts

**Figure 1:**
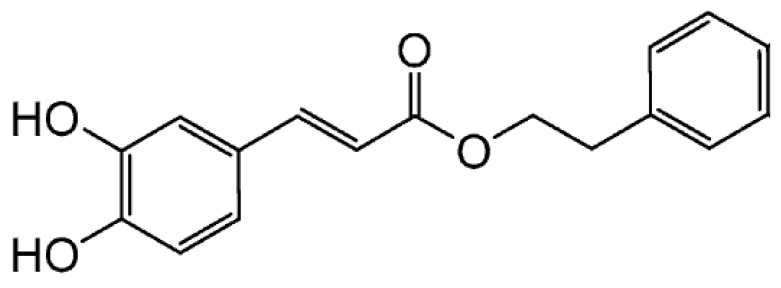
Chemical structure of caffeic acid phenethyl ester.

**Figure 2:**
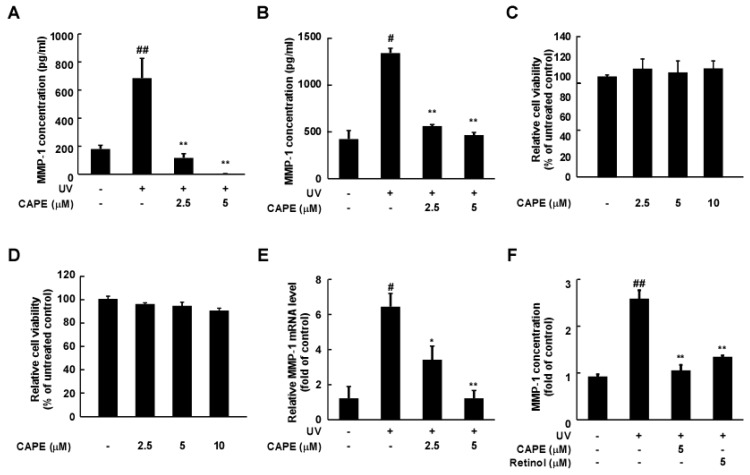
Effect of caffeic acid phenethyl ester (CAPE) on UV-induced MMP-1 in human dermal fibroblasts. Primary human dermal fibroblast (HDF) and Human foreskin fibroblast (Hs68) cells were pre-treated with CAPE for 1 h before being exposed to UV. (**A**,**B**) After 48 h, MMP-1 production in cultured media was measured using ELISA (*n* = 3). (**C**,**D**) Cell viability was measured after cells were treated with CAPE for 48 h (*n* = 4). (**E**) MMP-1 mRNA levels were determined using real-time PCR (*n* = 5). (**F**) MMP-1 production in cultured media was measured using ELISA (*n* = 3). The data are expressed as mean ± SD. ^#^ *p* < 0.05 and ^##^ *p* < 0.01 versus untreated control; * *p* < 0.05 and ** *p* < 0.01 versus the group exposed to UV alone.

To examine the anti-wrinkle effect of CAPE (Figure 1), we investigated MMP-1 production levels in cultured media, as MMP-1 is a secreted protein for collagen degradation. Two different dermal fibroblasts, Hs68 and HDF, were pre-treated with CAPE for 1 h and then exposed to UV irradiation. The UV exposure-mediated increase in the production of MMP-1 in the culture medium was significantly inhibited by CAPE treatment (2.5 and 5 μM) in HDF cells (Figure 2A) and Hs68 cells (Figure 2B). There were no significant differences in cell viability by CAPE in both type of cells (Figure 2C,D). To further examine whether CAPE-mediated inhibition of MMP-1 expression was occurring at a transcriptional or post-transcriptional level, the MMP-1 mRNA levels were evaluated after CAPE treatment. CAPE treatment significantly decreased the UV-induced MMP-1 mRNA levels (Figure 2E). Moreover, CAPE-mediated inhibition of MMP-1 displayed similar effects compared to that of retinol, a commonly used anti-skin wrinkle agent and well-known MMP-1 inhibitor [15] (Figure 2F).

### 2.2. CAPE Suppresses UV-Induced MMP-1 Expression in Human Skin Tissues

**Figure 3:**
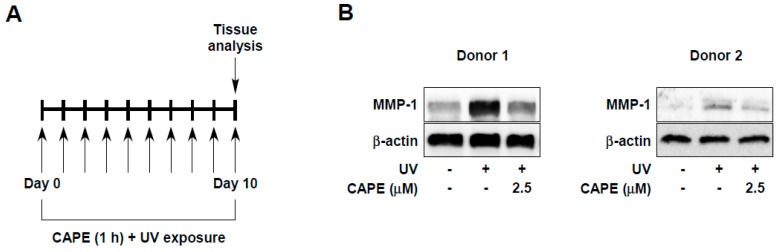
Effect of CAPE on UV-induced skin-wrinkle in ex vivo human skin tissue. (**A**) Human skin tissues were treated with CAPE at the indicated concentrations for 1 h, and then exposed to UV for 10 days. (**B**) MMP-1 protein expression was determined in human tissue lysate by immunoblotting. Skin tissues from two donors were used.

Next, we evaluated whether the effect of CAPE could be recapitulated in human skin. We analyzed UV-induced MMP-1 expression using ex vivo human skin tissues after CAPE treatment. Tissues were pre-treated daily with CAPE at the indicated concentrations, and then tissues were exposed to UV irradiation. After 10 days, MMP-1 levels were examined in the UV-irradiated skin tissue (Figure 3A). It was found that the 2.5 μM of CAPE treatment reduced MMP-1 expression in UV-irradiated skin tissues (Figure 3B). These results show that the preventive effect of CAPE against skin aging is observed not only in human skin cells but also in human skin tissues.

### 2.3. CAPE Inhibits UV-Stimulated Acetylation of Total Lysine and Histone H3 Lysine 9 in Both HDF Cells and Human Skin Tissues

**Figure 4:**
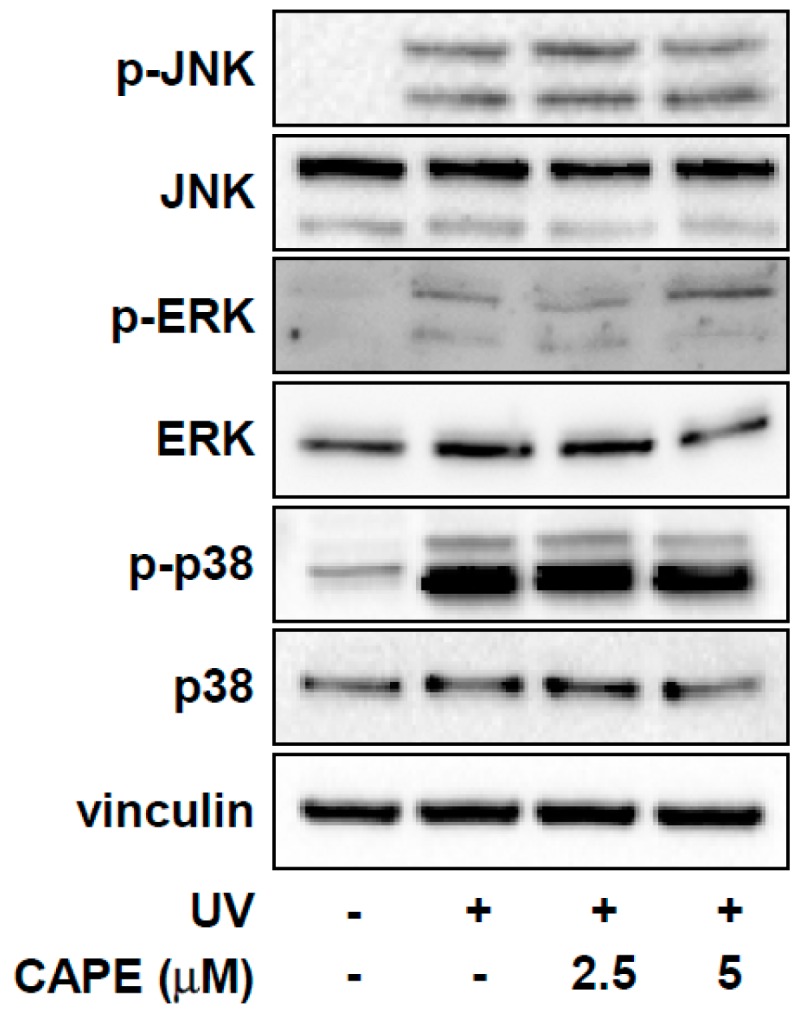
Effect of CAPE on UV-induced MAPK signal pathway in HDF cells. Cells were pre-treated with CAPE for 1 h before being exposed to UV. After harvesting the cells, phosphorylation and total protein expression of the MAPK signaling pathways were determined by immunoblot analysis.

The mitogen-activated protein kinase (MAPK) pathway including ERK, p38, and JNK, plays an important role in the regulation of MMP-1 expression [16]. We therefore investigated the effect of CAPE on MAPK signaling. However, CAPE (2.5 and 5 μM) did not exert any noticeable effect on the UV-mediated phosphorylation levels of p38, JNK, and ERK (Figure 4). These results suggest that the inhibitory effect of CAPE against UV-induced MMP-1 expression might be mediated by other mechanisms besides the MAPK pathway. 

**Figure 5:**
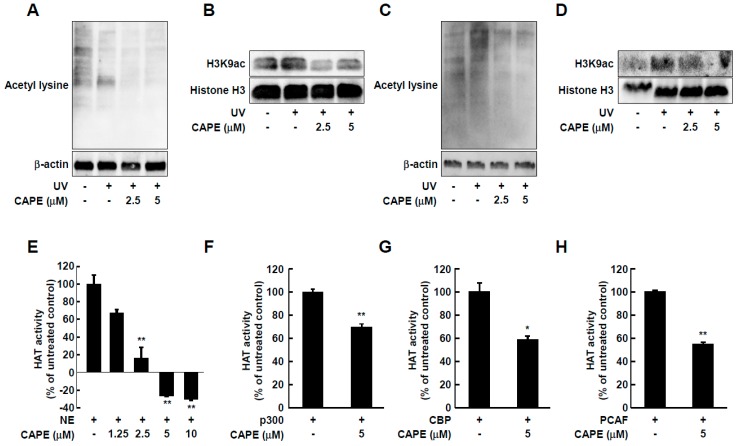
Effect of CAPE on histone acetyl transferase activity and UV-stimulated acetylated lysine and histone H3k9 acetylation in whole cell and tissue lysates. (**A**–**D**) HDF cells and tissue lysates were examined for the acetylation of lysine (non-histone) and histone H3 lysine 9 by immunoblotting. (**E**) Effect of CAPE on HAT activity was assessed in the presence of CAPE at the indicated concentrations using colorimetric HAT assay kit. (**F**–**H**) Enzyme-specific HAT activities of CAPE were measured using the purified enzymes p300, CBP, and PCAF. The data are expressed as mean ± SD of three independent experiments. * *p* < 0.05 and ** *p* < 0.01 versus untreated control.

Recently, epigenetic changes were found to participate in UV-mediated skin aging [5,17]. Specifically, induction of MMP-1 by UV is associated with epigenetic modification such as hyperacetylation of histones H3 [18]. To determine whether CAPE-mediated inhibition of MMP-1 involves epigenetic mechanisms, we evaluated the acetylation at lysine residues in total proteins as well as histones in cell and skin tissue lysates. CAPE treatment at 2.5 and 5 μM reduced UV-induced acetyl-lysine and acetylation of histone H3 at lysine 9 (H3K9ac), which is strongly associated with the UV-mediated induction of MMP-1 promotor [19] (Figure 5A,B). Consistent with the in vitro data, CAPE also attenuated total lysine acetylation and H3K9 acetylation in UV-stimulated human skin tissues (Figure 5C,D).

### 2.4. CAPE Acts as an HAT Inhibitor

To investigate whether the alteration in acetylation levels by CAPE was due to its effect on HAT, the HAT activity was measured in a cell-free system. As shown in Figure 5E, CAPE treatment significantly reduced HAT activity in a dose-dependent manner. More specifically, 5 μM of CAPE was able to inhibit the activity of p300, CREP-binding protein (CBP), and p300/CBP-associated factor (PCAF), which are representative types of HAT [20], suggesting that CAPE can act as a general HAT inhibitor (Figure 5F–H).

## 3. Discussion

In the current study, we have discovered that CAPE can suppress UV-induced MMP-1 expression in human dermal fibroblasts and in human skin tissues. The effect of CAPE was comparable to that of retinol, indicating the potential to be utilized as a novel agent for preventing skin aging. Investigation of the molecular mechanism led to the identification of HATs as the primary target of CAPE. In a previous study, CAPE showed anti-cancer effects on breast cancer by increasing histone H3 acetylation, working as an HDAC inhibitor [21]. Our study for the first time provides evidence that CAPE can directly suppress HAT activity and describes a new role for CAPE as an HAT inhibitor. 

Sun-exposed skin has been reported to have increased histone H3 acetylation in the promoters of *MMP-1, MMP-3, MMP-9, Ahr* receptor (collagen degradation), *PDCD5* (apoptosis), and *ITIH5* (inflammation). The solar irradiation-mediated alteration in histone acetylation profiles in the skin appears to be closely linked with changes in the expression pattern of histone modifying enzymes, such as upregulation of p300 or downregulation of HDAC1 and SIRT1 [9]. Several HAT inhibitors derived from natural products including genistein, curcumin, resveratrol, indole-3-carbinol, and epigallocatechin-3-gallate have been reported for the prevention and treatment of various human diseases [22]. Furthermore, HATs are known to be involved in the acetylation of not only histones but also transcription factors (non-histone proteins) [23], similar to what we have observed. Previous studies reported that an HAT inhibitor, anacardic acid (AA) blocked MMP-1 expression and histone modifications in HDF cells through suppressing p300 [8]. Furthermore, AA also inhibited UV-induced acetylation of histone H3, MMP-13, and COX-2 expression in both HDF cells and mouse skin tissue [8,24]. These studies indicate that epigenetic regulation via inhibition of p300 can be associated with protection from UV-mediated damages of the skin tissue. Our study demonstrates that CAPE can function as a novel HAT inhibitor to prevent skin aging by downregulating UV-mediated acetylation of histone and non-histone proteins in both human skin cells and human skin tissues.

## 4. Materials and Methods

### 4.1. Reagents

CAPE was purchased from Enzo Life Sciences (Farmingdale, NY, USA). Retinol was purchased from Cayman (Ann Arbor, MI, USA). Antibodies for phospho-p38, phospho-JNK, phospho-c-Jun, acetyl lysine, H3K9ac, and Histone H3 were purchased from Cell Signaling Technology (Beverly, MA, USA). Antibody for p38, JNK, c-Jun, and vinculin were purchased from Santa Cruz Biotechnology (Dallas, TX, USA). Antibody for MMP-1 was obtained from R&D systems (Minneapolis, MN, USA). 

### 4.2. Cell Culture and UV Irradiation

Human foreskin fibroblast (Hs68) and primary human dermal fibroblast (HDF) were cultured in DMEM (Corning Inc., Corning, NY, USA) containing 10% fetal bovine serum (FBS) (Gibco, Auckland, NZ, USA) with penicillin/streptomycin (Corning Inc., New York, NY, USA) at 37 °C in a humidified incubator containing 5% CO_2_. Hs68 cells were obtained from the American Type Culture Collection (ATCC, Manassas, VA, USA) and were cultured according to the ATCC’s recommended conditions. HDF cells were kindly provided by Dr. Jin-Ho Jung (Seoul National University, College of Medicine). Cells were irradiated with UV of 26 KJcm-2 for 26 min (called solar UV) using a solar UV light system (Q-Lab Corporation, Cleveland, OH, USA) in accordance with the method of the previous study [25]. The percentage of UVA and UVB from UVA-340 lamps was measured with UV meter at 94.5% and 5%, respectively.

### 4.3. Excised Human Skin and UV Irradiation

Human skin tissues, obtained from plastic surgery around the abdomen, were purchase from Biopredic international (Rennes, France). Briefly, the human skin tissues had been isolated from the abdomen after plastic surgery according to the French Law L.1245-2 CSP. Human skin provider, Biopredic International holds a permit (AC-2013-1754) granted by the French Ministry of Higher Education and Research for the acquisition, transformation, sales, and export of human biological material to be used in research. This study complied all principles set forth in the Helsinki Declaration. Tissues were incubated with high DMEM (Corning) containing 10% fetal bovine serum (Gibco) with penicillin/streptomycin (Corning) at 37 °C in a humidified incubator containing 5% CO_2_. Ex vivo human skin tissues were treated with CAPE and UV exposure repeated daily for 10 days (Figure 3A). 

### 4.4. Cell Viability Assay

Cell viability was determined using the Cell Titer-Glo assay Kit (Promega, Madison, WI, USA). Cells were seeded into white 96-well plates. After 24 h, the medium was replaced with serum-free DMEM and cells were treated with CAPE at the indicated concentrations and incubated for further 48 h. Cell Titer-Glo reagents were added to the cells. The plate was incubated for 20 min protected from light. Luminescence was measured on Varioskan Lux Multimode Microplate Reader (Thermo Fisher Scientific, San José, CA, USA).

### 4.5. Enzyme-Linked Immunosorbent Assay (ELISA)

Hs68 and HDF were seeded in 12 well plates at a density of 7.3 × 10^4^ cells/dish. After seeding, the cells were incubated for 24 h, after which the medium was replaced with serum-free media and incubated for another 24 h. Cells were treated with CAPE or retinol at the indicated concentrations and then irradiated with UV. Following 48 h of incubation, the culture supernatants were collected and centrifuged at 13,000 rpm for 2 min to remove the particulate matter and then stored at −80 °C in fresh tubes. The concentration of MMP-1 in the cell culture media was determined by corresponding ELISA kits (R&D systems Inc., Minneapolis, MN, USA). These assays were performed according to manufacturer’s instructions. In brief, the collected media were dispensed into a 96-well plate coated with human MMP-1 capture antibody. The plate was sealed and incubated at room temperature for 2 h. After the addition of MMP-1 detection antibody into each well followed by 3 washes, streptavidin-HRP was added. After washing the wells, substrate solution was added to the wells. After 20 min, the reaction was terminated using the 2N sulfuric acid solution. The absorbance was measured at 450 nm with a reference wavelength of 570 nm using the Varioskan Lux Multimode Microplate Reader (Thermo Fisher Scientific).

### 4.6. Real-Time PCR (qPCR)

Cells (1.7 × 10^5^ cells/dish) were seeded in 3.5 cm dishes. Total RNA was extracted from cells using NucleoSpin RNA isolation kit (Macherey-Nagel, Düren, Germany). cDNA was synthesized using a ReverTra Ace^®^ qPCR RT Master Mix with gDNA Remover (TOYOBO, Osaka, Japan). MMP-1 mRNA expression levels were analyzed using real time PCR using gene-specific primers. Primer sequences used were as follows: MMP-1 (Forward; GCATATCGATGCTGCTCTTTC, Reverse; ACTTTGTGGCCAATTCCAGG), GAPDH (FW; CCATCACCATCTTCCAGGAG, RV; ACAGTCTTCTGGGTGGCAGT). The reaction was carried out at 95 °C for 15 min followed by 60 °C for 60 min. qPCR was performed using Step One Plus Real-Time PCR System (Thermo Fisher Scientific) with THUNDERBIRD^®^ SYBR^®^ qPCR Mix (TOYOBO, Osaka, Japan) according to the manufacturer’s instructions. The quantitative value was normalized by GAPDH expression level. 

### 4.7. Immunoblot Analysis

Cells were washed twice with cold phosphate buffered saline (PBS) and lysed with RIPA lysis buffer containing protease and a phosphatase inhibitor cocktail (Sigma-Aldrich, St. Louis, MO, USA). The protein concentration was determined using a Pierce BCA Protein Assay Kit (Thermo Fisher Scientific). Equal amounts of total cellular proteins per sample were subjected to SDS-PAGE and transferred to a nitrocellulose (NC) membrane (PALL Corporation, Port Washington, NY, USA). The NC membrane was incubated with a specific primary antibody at 4 °C overnight. After incubation with HRP-conjugated secondary antibody, bands were detected with Western Lightning Plus-ECL (PerkinElmer, Norwalk, CT, USA). Protein bands were visualized using ChemiDoc^TM^ XRS+ System (Biorad, Hercules, CA, USA).

### 4.8. HAT Assay

The HAT activity of CAPE was confirmed using the HAT assay kit according to manufacturer’s protocol (Biovision Biotechnology, Militas, CA, USA). In brief, only HeLa nuclear extracts were used as a positive control. CAPE was prepared by concentration, and the total volume was set to 40 μL per well by ultra-pure water. Then, HAT substrate mixture, which included HAT substrates I and II, HAT assay buffer, and NADH-generating enzyme was added to each well. Plates were immediately read at 440 nm using a Microplate reader (Infinite 2000 PRO, Tecan, Männedorf, Switzerland). After incubation at 37 °C for 45 min, the absorbance was measured again at 440 nm. Specific HAT activity was measured using each enzyme (100 ng of p300, CBP, and PCAF) instead of nuclear extract.

### 4.9. Statistical Analysis

All data were analyzed with nonparametric methods using SPSS (Ver. 20; SPSS, Inc., Chicago, IL, USA) due to small sample sizes. Data are represented as mean ± standard deviation (SD). Statistical differences between mean values were determined by the Mann–Whitney test and Wilcoxon rank sum test between two groups, and a one-way ANOVA followed by post-hoc analysis with the Bonferroni test. A value of *p* < 0.05 was considered to indicate a statistically significant difference. 

## 5. Conclusions

The current study provides evidence for the first time that CAPE can block UV-stimulated MMP-1 levels in skin cells and in human skin tissues. The inhibitory effect of CAPE against MMP-1 appears to be associated with the inhibition HAT activity, leading to the attenuation of non-histone proteins (acetyl-lysine) and histone H3K9 acetylation. Therefore, CAPE as an HAT inhibitor may contribute to the prevention and treatment of skin aging caused by UV exposure.

## Abbreviations

CAPE	Caffeic acid phenethyl ester
UV	Ultraviolet
MMP-1	Matrix metalloproteinase
HDF	Human dermal fibroblasts
HAT	Histone acetyltransferase
CBP	CREP-binding protein
PCAF	p300/CBP-associated factor
HDAC	Histone deacetylase
MAPK	Mitogen-activated protein kinase
ERK	Extracellular-signal-regulated kinase
JNK	c-Jun N-terminal kinase
AA	Anacardic acid
COX-2	Cyclooxygenase-2

## References

[1] Debacq-Chainiaux F., Leduc C., Verbeke A., Toussaint O. (2012). UV, stress and aging. Dermato-Endocrinology.

[2] Fisher G.J., Kang S., Varani J., Bata-Csorgo Z., Wan Y., Datta S., Voorhees J.J. (2002). Mechanisms of photoaging and chronological skin aging. Arch. Dermatol..

[3] Orioli D., Dellambra E. (2018). Epigenetic Regulation of Skin Cells in Natural Aging and Premature Aging Diseases. Cells.

[4] Ho J.N., Lee Y.H., Park J.S., Jun W.J., Kim H.K., Hong B.S., Shin D.H., Cho H.Y. (2005). Protective effects of aucubin isolated from Eucommia ulmoides against UVB-induced oxidative stress in human skin fibroblasts. Biol. Pharm. Bull..

[5] Kammeyer A., Luiten R.M. (2015). Oxidation events and skin aging. Ageing Res. Rev..

[6] Shankar E., Kanwal R., Candamo M., Gupta S. (2016). Dietary phytochemicals as epigenetic modifiers in cancer: Promise and challenges. Semin. Cancer Biol..

[7] Peserico A., Simone C. (2011). Physical and functional HAT/HDAC interplay regulates protein acetylation balance. BioMed Res. Int..

[8] Kim M.K., Shin J.M., Eun H.C., Chung J.H. (2009). The role of p300 histone acetyltransferase in UV-induced histone modifications and MMP-1 gene transcription. PLoS ONE.

[9] Ding S., Chen J., Zeng Q., Lu J., Tan L., Guo A., Kang J., Yang S., Xiang Y., Zuo C. (2018). Chronic sun exposure is associated with distinct histone acetylation changes in human skin. Br. J. Dermatol..

[10] Shin S.H., Lee S.R., Lee E., Kim K.H., Byun S. (2017). Caffeic Acid Phenethyl Ester from the Twigs of *Cinnamomum cassia* Inhibits Malignant Cell Transformation by Inducing c-Fos Degradation. J. Nat. Prod..

[11] Chung T.W., Moon S.K., Chang Y.C., Ko J.H., Lee Y.C., Cho G., Kim S.H., Kim J.G., Kim C.H. (2004). Novel and therapeutic effect of caffeic acid and caffeic acid phenyl ester on hepatocarcinoma cells: Complete regression of hepatoma growth and metastasis by dual mechanism. FASEB J..

[12] Jung W.K., Lee D.Y., Kim J.H., Choi I., Park S.G., Seo S.K., Lee S.W., Lee C.M., Park Y.M., Jeon Y.J. (2008). Anti-inflammatory activity of caffeic acid phenethyl ester (CAPE) extracted from *Rhodiola sacra* against lipopolysaccharide-induced inflammatory responses in mice. Process Biochem..

[13] Kudugunti S.K., Vad N.M., Ekogbo E., Moridani M.Y. (2011). Efficacy of Caffeic Acid Phenethyl Ester (CAPE) in skin B16-F0 melanoma tumor bearing C57BL/6 mice. Investig. New Drugs.

[14] Murtaza G., Karim S., Akram M.R., Khan S.A., Azhar S., Mumtaz A., Bin Asad M.H. (2014). Caffeic acid phenethyl ester and therapeutic potentials. BioMed Res. Int..

[15] Seong J.S., Xuan S.H., Park S.H., Lee K.S., Park Y.M., Park S.N. (2017). Antioxidative and Antiaging Activities and Component Analysis of *Lespedeza cuneata* G. Don Extracts Fermented with Lactobacillus pentosus. J. Microbiol. Biotechnol..

[16] Pittayapruek P., Meephansan J., Prapapan O., Komine M., Ohtsuki M. (2016). Role of Matrix Metalloproteinases in Photoaging and Photocarcinogenesis. Int. J. Mol. Sci..

[17] Pollack B.P., Sapkota B., Boss J.M. (2009). Ultraviolet Radiation-Induced Transcription is Associated with Gene-Specific Histone Acetylation. Photochem. Photobiol..

[18] Kim M.K., Lee D.H., Lee S., Kim E.J., Chung J.H. (2014). UV-induced DNA damage and histone modification may involve MMP-1 gene transcription in human skin In Vivo. J. Dermatol. Sci..

[19] Ujfaludi Z., Tuzesi A., Majoros H., Rothler B., Pankotai T., Boros I.M. (2018). Coordinated activation of a cluster of MMP genes in response to UVB radiation. Sci. Rep..

[20] Roth S.Y., Denu J.M., Allis C.D. (2001). Histone acetyltransferases. Annu. Rev. Biochem..

[21] Omene C., Kalac M., Wu J., Marchi E., Frenkel K., O’Connor O.A. (2013). Propolis and Its Active Component, Caffeic Acid Phenethyl Ester (CAPE), Modulate Breast Cancer Therapeutic Targets via an Epigenetically Mediated Mechanism of Action. J. Cancer Sci. Ther..

[22] Shankar S., Kumar D., Srivastava R.K. (2013). Epigenetic modifications by dietary phytochemicals: Implications for personalized nutrition. Pharmacol. Ther..

[23] Masumi A. (2011). Histone Acetyltransferases as Regulators of Nonhistone Proteins: The Role of Interferon Regulatory Factor Acetylation on Gene Transcription. BioMed Res. Int..

[24] Kim M.K., Lee S., Kim E.J., Kong K.H., Lee D.H., Chung J.H. (2013). Topical application of anacardic acid (6-nonadecyl salicylic acid) reduces UV-induced histone modification, MMP-13, MMP-9, COX-2 and TNF-alpha expressions in hairless mice skin. J. Dermatol. Sci..

[25] Han A., Lee J., Lee M.H., Lee S.Y., Shin E.J., Song Y.R., Lee K.M., Lee K.W., Lim T.G. (2019). Sulfuretin, a natural Src family kinases inhibitor for suppressing solar UV-induced skin aging. J. Funct. Foods.

